# In This Issue

**DOI:** 10.1002/cfg.204

**Published:** 2002-08


**Comparative and Functional Genomics**
*Comp Funct Genom* 2002; **3**: 305.
Published online in Wiley InterScience (www.interscience.wiley.com). **DOI:** 10.1002/cfg.204

### In this issue

### Heterologous array analysis of pinaceae

Leonyl van Zyl *et al.* report on a hybridization of *Pinus taeda* cDNA arrays with cDNA from needles and embryogenic cultures of *P. taeda*, *P. sylvestris* and *Picea abies*. Since the level of sequence conservation between *Pinus* and *Picea* is high, they investigated the use of arrays from one genus for studies of gene expression in the other. Their data shows that arrays of cDNA from loblolly pine are useful for studies of gene expression in other pines or spruces.

### Harvesting the mouse genome

The international Mouse Sequencing Consortium has recently announced a public draft of the mouse genome, and the mouse sequencing milestones have been brought forward. Marc Botcherby presents a timely overview of the current status of mouse genome sequence and mapping resources.

### Special Section on the 1st Symposium of the Wellcome Trust funded Multi-Collaborative Microbial Pathogen Microarray Facility: BμG@S 2002

The editor of this section, Philip Butcher, introduces the BμG@S facility, and the symposium, in his editorial, which is followed by a discussion on 'Microarrays for bacterial pathogens: Hope or hype' by Brendan Wren.

### Conference Reviews

— **Hinds *et al.*** detail the BμG@S approach to pathogen microarray design and production.
— **Dorrell *et al.*** discuss their microarray analysis of *Campylobacter jejuni*.
— **Inwald *et al.*** report on their microarray-based comparative genomics of *Mycobacterium bovis* spoligotypes.
— **James *et al.*** present an approach to profiling *in vitro* gene expression using chemostat culture for *M. tuberculosis*.
— **Stewart *et al.*** have used array analysis to characterise the heat shock response of *M. tuberculosis*, and have analysed deletion mutants to dissect this important stress response.
— **Kendall *et al.*** review how microarray analysis of bacterial gene expression can allow us to move towards an understanding of the bacterial regulome.
— **Hinchliffe *et al.*** present a microarray analysis of two strikingly similar *Yersinia* species, *Y. pestis* and *Y. pseudotuberculosis*.
— **Ali *et al.*** discuss how *H. influenzae* microarrays can lead to a better understanding of virulence and aid the search for vaccines.
— In their analysis of strains from a recent TB outbreak Shafi *et al.* have shown that microarrays can be a rapid and powerful tool in the study of the genomic epidemiology of *M. tuberculosis* in clinical isolates.
— **McCluskey *et al.*** review the progress they have made so far in applying microarray technology to the analysis of *S. pneumoniae*.
— **Witney and Hinds** describe BμG@Sbase: a microarray database and tools (currently under development) for supply to for the BμG@S user community.
— **Lorenz Wernisch** asks 'Can replication save noisy microarray data?' He looks at ANOVA mixed models and analysis of variance components as a rigorous way to work out the required number of replicates to detect a given fold-change in expression.
— In 'The curse of normalisation', **Wolkenhauer *et al.*** discuss some of the difficulties encountered when analysing microarray data.

### Meeting Review: Array Bioinformatics

**Douglas Roy *et al.*** report on 'Bioinformatics of Biochips: Accelerating Discovery in Functional Genomics', a research workshop organised by members of the Scottish Centre for Genomic Technology and Informatics.

Copyright © 2002 John Wiley & Sons, Ltd.

